# Social capital and health during pregnancy; an in-depth exploration from rural Sri Lanka

**DOI:** 10.1186/s12978-017-0349-7

**Published:** 2017-07-27

**Authors:** Thilini Chanchala Agampodi, Thilde Rheinländer, Suneth Buddhika Agampodi, Nicholas Glozier, Sisira Siribaddana

**Affiliations:** 1 Department of Community Medicine, Faculty of Medicine and Allied Sciences, Rajarata University of Sri Lanka, Saliyapura, Sri Lanka; 2 Department Of Public Health, Global Health Section, University of Copenhagen, København, Denmark; 3 Brain and Mind Research Institute & Discipline of Psychiatry, Sydney Medical School, The University of Sydney, Camperdown, Australia; 4 Department of Medicine, Faculty of Medicine and Allied Sciences, Rajarata University of Sri Lanka, Saliyapura, Sri Lanka

**Keywords:** Sri Lanka, Social capital, Health, Pregnancy, Qualitative

## Abstract

**Background:**

Dimensions of social capital relevant to health in pregnancy are sparsely described in the literature. This study explores dimensions of social capital and the mechanisms in which they could affect the health of rural Sri Lankan pregnant women.

**Methods:**

An exploratory qualitative study of solicited diaries written by pregnant women on their social relationships, diary interviews and in-depth interviews with key informants was conducted. A framework approach for qualitative data analysis was used.

**Results:**

Pregnant women (41), from eight different communities completed diaries and 38 post-diary interviews. Sixteen key informant interviews were conducted with public health midwives and senior community dwellers. We identified ten cognitive and five structural constructs of social capital relevant to health in pregnancy. Domestic and neighborhood cohesion were the most commonly expressed constructs. Social support was limited to support from close family, friends and public health midwives. A high density of structural social capital was observed in the micro-communities. Membership in local community groups was not common. Four different pathways by which social capital could influence health in pregnancy were identified. These include micro-level cognitive social capital by promoting mental wellbeing; micro-level structural social capital by reducing minor ailments in pregnancy; micro-level social support mechanisms promoting physical and mental wellbeing through psychosocial resources and health systems at each level providing focused maternal care.

**Conclusion:**

Current tools available may not contain the relevant constructs to capture the unique dimensions of social capital in pregnancy. Social capital can influence health during pregnancy, mainly through improved psychosocial resources generated by social cohesion in micro-communities and by the embedded neighborhood public health services.

## Plain English summary

Social capital, which simply means the state of social relationships of individuals and communities, is recognized as a major determinant of health; however, studies on social capital and maternal health are scarce. Even in Sri Lanka, where coverage of maternal health services is high, the situation remains same. The aim of this study was to describe the state of social relationships and their effects on health during pregnancy. A detailed qualitative study, including diaries written by pregnant women, diary interviews and in-depth interviews with primary health care providers and senior community members were conducted. The results of this study were able to identify a variety of social relationships (dimensions of social capital) that are relevant to the health of pregnant women. Mechanisms in which social capital could influence maternal health, were also identified. The findings of this study may help to improve the validity of the measurement of social capital in pregnancy and assist in planning interventions to improve health of pregnant women by cultivating social capital.

## Background

Social determinants are a major underlying cause for inequities in health [1]. The World Health Organization (WHO) Commission for Social Determinants of Health, in 2005 undertook the task of describing the social determinants, observing how they operate and suggesting how to manipulate them to reduce health inequities [2]. Emerging as a factor among the social determinants of health, social capital, has been extensively studied in high income countries (HIC) [3–7]. In contrast, it has not been a popular theme in the health agenda of low and middle income countries (LMICs) [8, 9].

Sri Lanka has achieved exemplary progress in maternal health in the past century [10]. The maternal mortality rate is low (32.5/100,000 live births) [10], compared to other LMICs and the country has 99–100% coverage in antenatal care. This success has been attributed to a strong public health network, promotion of institutional deliveries (99.9%) [10], free preventive and curative care services, high female literacy level and a favorable culture that provides care for pregnant women. Nevertheless, a further reduction of maternal mortality has been a challenge; one of the neglected areas has been social aspect of health during pregnancy.

Pregnancy is an emotionally sensitive period in the life of a woman mainly due to the hormonal effects. Women may also become socially vulnerable, vulnerable to minor ailments and have reduced productivity [11], demanding the need for extra care and support during pregnancy. While social re-arrangements such as improving social participation could improve maternal health [1], the global maternal health agenda is still focusing mainly on essential interventions to reduce maternal mortality [12]. The few studies available on social capital and maternal health, show that social support and social networks are associated with better self-rated health in pregnancy [13] and strong social networks are associated with improved pregnancy outcomes [14, 15].

Despite its wide use, social capital has been a subject of debate over the past few decades [5, 16–19]; as a result, there are many definitions for social capital. Bordieu in 1986, provided a theoretically refined definition [17]; defining social capital as the “aggregate of the actual or potential resources which are linked to possession of a durable network of more or less institutionalized relationships of mutual acquaintance or recognition”. Putnam defined social capital as “features of social organization, such as trust, norms and networks that can improve the efficiency of society by facilitating coordinated actions” [20]. In 1990, Coleman defined social capital according to its function; “social capital is not a single entity, but a variety of different entities having two characteristics in common: they all consist of some aspect of social structure, and they facilitate certain actions of individuals who are within the structure” [18]. “Relationships”, “norms”, “institutions” and “networks” are the most common constructs used to define social capital.

Understanding the theoretical framework of social capital would be beneficial in study planning and interpretation of results. In 1993 Robert Putnam viewed social capital in its narrow form of horizontal associations [20]. This included micro-level relationships such as family and neighborhood relationships and membership in groups. Coleman extended this concept to include meso-level civic engagement, which comprised of not only horizontal networks but vertical as well [18]. Later, social capital was viewed in the broader socio-political context and the impact of macro-level organizational structure of social affairs was given more priority especially in concern with the economic development [21].

Social capital has two major dimensions. Structural social capital refers to externally observable objective aspects of social organization [22–24]. Cognitive social capital is subjective and consists of the norms, values, attitudes and beliefs of people that affect social participation and mutual support [5]. Structural and cognitive dimensions of social capital can be complementary [21]. Szreter and Woolcock developed the most recent framework for social capital and public health [25]. This framework includes three aspects in which social capital could influence population health; the “social support” perspective, “inequality theses” and “political economy” approach. They distinguished social capital in the three forms; “bonding”, “bridging” and “linking”. Bonding refers to strong ties of trust and co-operation between close individuals such as family members, close friends, relatives and neighbors [23]. Bridging refers to week ties between individuals considered different, such as people from different ethnic groups but within the same level in terms of status of power [22]. The social support perspective and inequality theses were included within these two dimensions. Linking describes the relationships among people with different hierarchies of power or authority, which would explain the influence of political economy approach [26].

Social capital is context dependent. There is a gap in the published literature in describing the dimensions of social capital relevant to pregnancy. Most studies have only measured social support and networks [15, 27]. A systematic review indicated that other major constructs of social capital such as social trust, sense of belonging and social cohesion are more associated with health than social support and networks in LMICs [9]. Measurement of social capital is complex and there is no gold standard tool. Different constructs of social capital, relevant for the study population, are used to interpret a social capital measure. Some investigators have used secondary data from large population surveys [9] while some have constructed composite tools using primary data [28]. However, there is no specific tool developed to measure it in pregnant women, possibly because there haven’t been in-depth qualitative studies to explore and identify the relevant constructs.

The influence of social capital on maternal health is debated, some argue it is due to creation of better socio-economic circumstances (“inequality theses” [25]) or due to better psycho-social support (“social support mechanism” [25]) to reduce life stressors [15]. It is also important to find how socio-political context could affect the health during pregnancy. We argue that inductive qualitative studies would be best to identify the grass roots level links and mechanisms of this framework. It would be valuable to identify these mechanisms in order to facilitate effective social re-arrangements to improve maternal health.

To date, there are no studies investigating the nature of social capital of Sri Lankan pregnant women. This study aims to explore social capital of rural Sri Lankan pregnant women, identifying the social capital dimensions in pregnancy and to describe the mechanisms of how social capital would affect health during pregnancy.

## Methods

This study is a part of a larger study conducted for cultural adaptation and validation of a tool to measure social capital related to health among pregnant women. The detailed study protocol for the present study is published elsewhere [29], therefore, only a brief description of methods is presented here.

### Study design

An exploratory qualitative design was used. Initially social capital in pregnancy was explored through solicited diaries of 41 pregnant women. The data from the diaries were followed with 38 interviews. Subsequently, 16 in-depth interviews with Public Health Midwives (PHMs) and senior community dwellers were conducted.

### Study setting and population

The study was conducted in Anuradhapura, the largest district in North Central Province of Sri Lanka with a population of 886,945. In this district, 94.1% of the population is rural [30]. The main stay of economy is agriculture. There are 90.7% Sinhalese, 0.8% Tamil and 8.13% Sri Lankan Moor ethnic groups in this district [30]. More than 19,000 pregnant mothers are registered annually for public antenatal care in Anuradhapura [10]. According to the Demographic Health Survey (DHS), 90% of females in the district have completed secondary school [31].

### Selection of study sample

#### Selecting communities

**Fig. 1:**
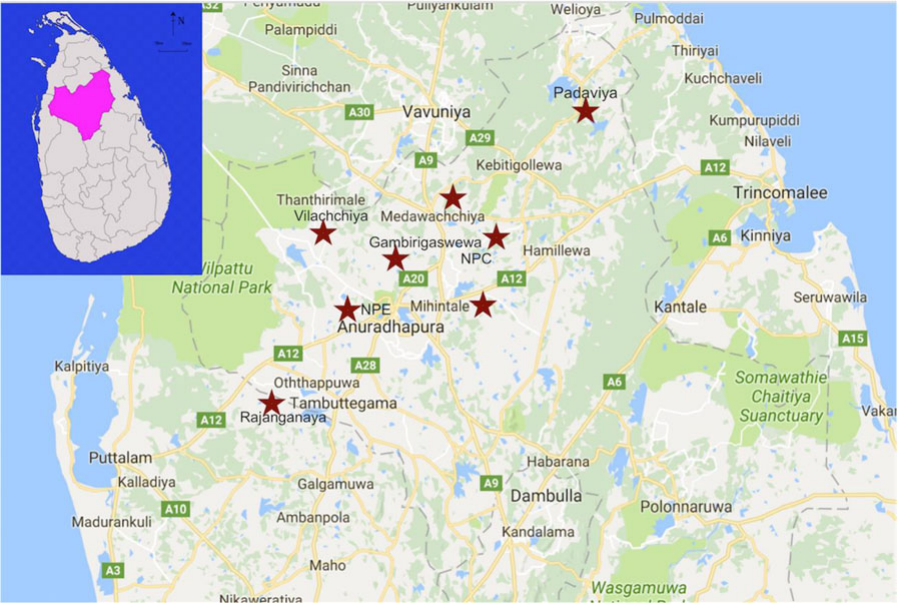
Map of Anuradhapura district, Sri Lanka showing the communities selected for the study

To reflect the diversity within the district, eight different types of communities were included, which were identified through informal discussions with medical officers of health (MOH) and other key informants such as public health nursing sisters (PHNSs) and PHMs (Fig. 1). Communities were semi-urban (NPE), and seven were rural. These rural communities included an agrarian resettled community under a major irrigation project (R), an “ancient village” community (Me) where generations from ancestors of the Sri Lanka resides, a conflict affected community (P), an ethnic minority (Moor) community (GA), another two rural communities in which socio- demographic characteristics and health seeking behaviors of people are known to be different from the general rural population (V, Mi) and a community to represent the general rural population (NPC). The letter abbreviations (which were used for coding purposes) represent the names of the communities selected.

#### Selecting study participants

We identified and recruited participants in each community group through the public health midwives (PHMs). The total study period extended to 8 months, 1 month for each community. PHMs are the grass root level public health service provider for maternal and child health in Sri Lanka. The PHM in each community selected five to seven pregnant women in different gestational periods, with different educational levels and socio-economic backgrounds. There were no restrictions in age, ethnicity or educational level (almost all women had completed above primary education in Sri Lanka). Women residing in the area for the past 3 months were included. Additional participants were selected up to the point of data saturation.

One PHM and one community dweller in each community were selected for in-depth interviews as key informants.

### Study instruments

#### Phase 1

##### Participant diaries

Participant diaries have been used in studies regarding pregnancy to acquire data on physical activity [32]. The diaries used in this study was prepared according to the available guidelines in literature [33]. An A4 30-page booklet was used. An information sheet on the purpose of the diary, a list of open-ended questions to guide the process, and instructions was provided on the first page [29]. An example completed diary page was provided on the second page. An additional page was provided at the end to document the participant’s comments. Diaries were immediately collected at the end of the period of diary writing.

##### Diary interviews

Following 2 weeks of daily diary entries, investigators reviewed the diaries and prepared. The interviews took place within 2 weeks of completing diary writing. Trained pre-intern medical officers conducted the interviews. Each face-to-face interview lasted around 20–40 min. They were done either at the participants’ residence or at antenatal clinics. All interviews were tape recorded with the consent of the participants.

#### Phase 2

##### In-depth interviews with key informants

In *phase 2,* in-depth interview guides were developed based on the diary data to elaborate on views of social capital in pregnancy from the eight selected senior community representatives and eight PHMs.

### Data collection procedure

Pregnant women were instructed to document their social lives for 2 weeks, which has been argued as the optimum duration for diaries of this nature [34]. The participants were contacted through mobile phones 2–3 times during this period, to remind them to diary and to clarify any doubts. Two women could not be followed by phone, however, they provided their diaries at the end of 2 weeks. One participant terminated participation early due to illness. Three participants did not complete diary interviews due to changes in residence, inconvenience to participate after delivery and difficulty in contacting them. The participants were also given the opportunity to contact the principal investigator if required clarification.

The principal investigator and five pre-intern medical graduates (three female and two male), who were trained on qualitative methods, research ethics, respecting the traditional practices of different communities, conducted all interviews. The study tools were pilot tested in a community where the study was not being carried out and were adjusted accordingly. In-depth interviews were conducted according to Family Health International (FHI) guidelines [35] and other relevant texts available in literature [36]. These guidelines were used for training, formulating guides and checklists, developing note taker forms, conducting interviews and the storage of data.

Each diary and in-depth interview recording was transcribed verbatim. An investigator fluent in both English and the Tamil language translated the diaries, diary interviews and the in-depth interview recordings and notes of women from the ethnic minority (moor) community.

### Data analysis

A framework approach for qualitative data analysis was employed [37]. The framework approach, instead of thematic analysis, was used because there was preexisting set of dimensions and constructs for social capital in literature. The framework was prepared using the most recent classification of major dimensions of social capital (cognitive, structural and bonding, bridging and linking) [38]. The constructs for each dimension were selected from documented best tools for LMICs that were identified from a previous systematic review [3, 23] (Column one; Tables 1 and 2). Defining the constructs were done according to available literature and on consensus of all authors [39–48]. The following steps for framework analysis were followed; familiarization of the data, developing a framework, coding the data, matrix formation and interpretation. During the analysis any emerging additional constructs of social capital discovered were added to the framework, with the consensus of all investigators.

**Table 1:** Framework analysis of cognitive constructs (positive and negative) of social capital in pregnancy

Social capital construct	Definition	Descriptors	Level & type of social capital	Selected verbatim from diaries	Selected verbatim from PHM/CD interviews
Domestic cohesion (husband)^a^	The emotional bonding that family members have towards one another and the degree of individual autonomy an individual experience in the family system (Oslon et al. 1979 [53])	Feel happy when the husband is around	*Individual Bonding*	“My husband was at home today. I love it so much when my husband is around. It is the best of satisfaction I could imagine.” Me04	“Most husbands are not at home. They work outside the village and visit home around once a month. When they are at home, they are very helpful to mothers.” Me Community Dweller (CD)
		Being cared by the husband	*Individual Bonding*	“My husband take care of me now more than previously. He bought me a garden hose, as it was difficult for me to water the garden. He fixed it and I was very happy.” Mi 02	“Husbands are very concerned about their wives during pregnancy. Sometimes they call us even in the night asking for advise on their wives symptoms.” NPE PHM
		Husband is providing care when ill	*Individual* *Bonding*	“I felt ill at around 1.00 P.M So I couldn’t conduct evening class. My husband suggested that we should take treatment. But I refused to go, as I’m pregnant and I shouldn’t take more drugs. He stayed closer to me & looked after me with kindness. I got the feeling that his kindness cured me.” GA01	Husband pay lot of attention on health of the wife during pregnancy. They are very supportive. When we visit the house, it is the husband that prepares and offers us refreshments while we are talking to the mother. GA PHM
		Express love to husband	*Individual* *Bonding*	“When my husband is at home, I never feel alone. He is like a god to me. I feel that I’m the most fortunate wife in the world!” R04	
		Disharmony and conflicts with husband	*Individual*	“My husband came home and asked me for money. I asked why? He told that it is to get liquor. I was very angry and scolded him. He got angry too and slept. I felt very sad as to why he does this to us while we are struggling to overcome our problem.” V02	“Most of the husbands in this community do security jobs out of town. Therefore the relationship between the husband and the wife is poor. The love and care received by the females during pregnancy is low in this community. It is very difficult to get the husband to attend an antenatal session or a monthly weighing program of a child.” V PHM
Domestic cohesion (Family)^a^		Trust towards in-laws	*Domestic* *Bonding*	My mother-in-law is very kind. She will not react even when we do something unreasonable for her. Such a good person she is! Better than my own mother. My Father in-law is the same. My sister in-laws never let my husband scold my children or me. In such situations they confront him. P01	Most of the time mother-in-laws take care of pregnant daughter-in-law as their own daughter. Conflicts are seen rarely when the daughter-in-law comes from an area with relatively higher socio-economic facilities is resistant to adjusting to this community. P PHM
		Family members spend time talking together	*Domestic* *Bonding*	“Evening was joyful. We were all sitting in the veranda and were teasing my sister-in- law. All of us were laughing. I forget my ailments when she says funny things..” NPC01	
		Family members work together	*Domestic* *Bonding*	“As today is Friday, I came home at around 11.00A.M. Then my mother-in-law, sister-in-law & myself prepared the lunch” GA01	
		Family members are concern of the arrival of newborn	*Domestic Bonding*	“Everybody in my house are awaiting for the great day! I’m very happy that my child is gifted with such a lovely family. The child is very lucky” NPC01	
		Feel cared at home when ill	*Individual Bonding*	“Today I had severe pain in my upper arm. My mother-in-law & sister-in-law did all household work. They applied medicines to my arm. They prepared food. I can’t forget their help.” GA 01	“This is a closely knit community. Usually the extended family live with the pregnant women and they take care of her.” GA PHM
		Disharmony with in-laws	*Domestic* *Poor bonding*	“Some of my husbands’ relatives are jealous of our love. I have no close relationship with my mother in-law. I talk what is necessary only. My mother-in-law’s marriage is not successful. Therefore they don’t like us to be happy.” R03	“There are problems with in-laws. There’s a generation gap. Pregnant daughters don’t want to listen to their mother-in-law. They say that she is old. Therefore there is disharmony in the family.” R PHM
		Newborns arrival perceived as a burden	*Domestic* *Poor bonding*	“When there are problems at home I feel angry all the time. It’s difficult for me to control my self. The main reason is my unborn child. My husband likes to have it, but not my elder children. They don’t like me becoming pregnant. It’s difficult for me to work. My whole freedom is gone. I have to do the whole stuff again.” V04	
Neighborhood cohesion^a^	The tendency for a group to be in unity while working towards a goal or to satisfy the emotional needs of its members (Carron, A.V., Brawley, L.R. (2000).	Feel cared by the neighbors	*Individual Bonding*	“Every one in the neighborhood loves and cares about me.” NPC01	
		Like spending time with neighbors/ relatives/friends	*Individual* *Bonding*	“After preparing the dinner I went to the next door aunts’ house as I felt lonely and then to a friends house as well. I was able to get rid of the boredom. I was relieved.” Me04	
		Tolerance of diversity of neighbors	*Neighborhood* *Bonding*	“I also take another child in the neighborhood when I take my child to school in a three wheeler. This child always try to provoke by pulling up things of my soul daughter. I advised my child not to react. I’m happy that my child still loves this child despite her irritating behavior.” NPE01	
Neighborhood trust^c^	Belief in the honesty, integrity and reliability of neighbors (Taylor P., Funk C. 2010)	Perceive neighborhood as a favorable place to adopt the child	*Neighborhood* *Bonding*	“I feel that this neighborhood is the best to bring up my child. When people around are generous my child will see it and hear good things always” NPC01	
Neighborhood mistrust^c^	Have doubt or suspicion of neighbors	Refrain from visiting or talking to neighbors (mistrust)	*Neighborhood* *Lack of bonding*	“Although I feel alone at home, I don’t have the habit of going around to neighboring houses and spending time talking to them. Most of the people are relatives. But one should be careful of speaking to them. They utter slander. Nonrelated people help me more than my relatives.” R04	“When there’s a problem mothers come and tell us. They don’t want other people to hear it. They can get others support if they do so. But it does not happen here. There is some what mistrust in the neighborhood.” R PHNS
Reciprocity^a^	Actions that are contingent on rewarding reactions from others and that cease when these expected reactions are not forthcoming. (Balu P.M. 1964)	Neighbors help each other	*Neighborhood Bonding*	“My husband has gone for a course in “Ampara” district (Far away from this village). His family is not happy with me. I only have my neighbors to share my joy and sorrow. Therefore I keep in harmony with all my neighbors. They help me a lot. Today they brought me lunch” V01	
Informal social control^c^	Reactions of individuals and groups that brings about conformity to norms and laws. (Coklin J 2007)	People in the community give priority for pregnant women	*Community* *Bonding& bridging*	“I went to meet the village leader today. I didn’t have to wait in the queue, as I’m pregnant. Work get done easily” NPC02	Usually when women become pregnant the villagers take care of her. They often offer food to a pregnant woman when they prepare a special meal. Even at social gatherings people give priority to pregnant women. V PHM
		Concerned about other people	*Community* *Bridging*	“I was asked to play the organ at the chapel but I told that I will do it next week as it will hurt feelings of the child who came to play it today” NPE02 “When my neighboring aunt was admitted to hospital, everyone in the village went to see her.” NPC01	
Sense of belonging^b^	Feeling of acceptance as a member of a group (Yosephine E. 2014)	Receive complements on birthday	*Individual* *Bonding*	“Today is my birthday! My husband, family members and relatives wished me good luck.” NPC01	
		Being known by the villagers	*Individual* *Bridging*	“I have a fair reputation in the village. I’m an outsider and many do not know me. But all of them know my husband.” NPC01	
Loneliness^b^	Distressing feeling that occurs when ones social relationships are perceived as being less satisfied than what is desired. (Cacioppo J. T.,,Hawkley L. C. 2012)	Feel lonely at home	*Individual*	“Today was a very bad day for me. Nobody visited us and we didn’t go anywhere. My mother-in-law and myself stayed home alone. I was very lonely” NPC 03	
		Misses own family/relatives	*Individual* *Lack of bonding*	“I came home from the clinic around 12.00 pm. I was so tired and unwell. At that time I remembered my own mother. If I was with her she will take care of me very well. I’m staying with my in-laws. I don’t receive the love and care that I would have received if Iwas with my mother.” R03	
Social support^a^	System of formal and informal relationships through which individuals receive emotional support, material or information to cope with stressful emotional situations (Caplang G. 1974)	Have support on household chores (instrumental support)	*Individual*	“I came home around 11.30 A.M from the nursery. I felt tired. My mother-in-law and sister-in-law had finished preparing lunch by that time. So I was able to have a nap. As I’m pregnant most of the time they do the household work.” GA01	
		Have a close person to share feelings (emotional support)	*Individual*	“A very close person to me visited our place today. It was a great pleasure to see her. She is an aunt whom I share my joy and misery with. She has brought dinner for me too” NPC02	
		Have a person to provide financial support when needed (Instrumental support)	*Individual*	“Although I felt reduced fetal movements, we did not have money to see a specialist doctor. But when I told this to my mother she gave me money which was a great relief” NPC06	
		No one to care when ill (Lack of instrumental support)	*Individual*	“There’s no one to inform when I fell ill at night. Cannot call my husband either as he sleeps after the days work. At such times I grab my small one towards me and shut my eyes tight!” R04	
		No one to accompany in going out	*Individual* *Lack of bonding*	“I had to go for the ultra sound scan today. My number was ten. So I asked my mother-in law to get ready for the bus leaving at 12.10 pm. She told that she couldn’t come today, as she has to go to her own daughters place. Then I went to see whether my relative neighbor aunt was available. But she was not at home. I made my mind to go alone. What else to do, its for my child’s sake..” V01	
		Lack of support on house hold chores (Lack of instrumental support)	*Individual*	I’m struggling to work on my own. At this stage, it is difficult for me to do anything. Therefore I keep thinking a lot every day. It is with lot of difficulty that I brought up my elder two. Because of this new child I won’t be able to help the other two with their school work. I have to look into everything in he family on my own. There is no one to help. If I were not pregnant I would have cultivated and obtained electricity for the house. V04	“It varies among families. Some times the husband thinks only of his satisfaction. Total responsibility on maintaining the family is given to the wife. But sometimes both work as one.” V CD
Social responsibility/ contribution^c^	An individuals obligation to act for the benefit of the society (Sreenivasulu 2013)	House hold responsibility	*Individual*	“I woke up in the early morning as I had to wake up my child for his studies. He has become very lazy. It was difficult to wake him up. I made tea and while helping his studies prepared the breakfast. At 6.45 am, I sent him to school because his schoolwork starts at 7.00 am. I have to feed him make his clothes and help him to get ready. Otherwise he will leave without having a breakfast.” V04	“There are two types of mothers. Some are like babies and husband and family provide everything for them. But some holds the total responsibility on household and adopting young ones.” V PHM
		Take responsibility in religious activity in the neighborhood	*Individual*	The prize-giving of the drawing competition in the church went well. Although it interrupted teaching at the Sunday school, organizing it gave me satisfaction. NPE01	
		Responsibility in education of young	*Individual*	“In the evening I taught my younger sister.” ME04	
		Hold responsibility in village organizations	*Individual*	“I have developed more relationships in my second pregnancy than in my first pregnancy. Because I did 1 year diploma in women empowerment & after that with the help of PHM we started “mothers’ club”. I had several meetings with pregnant women not only our village, but also in our neighboring village with the help of PHM. We advised them regarding pregnancy. With that I was happy & I lost sadness, laziness & fear. Instead leadership & courage was with me.” GA03	
Trust in institutions^a^	How people perceive how well the institutions are operating (Miller 1974)	Health systems	*Individual*	“At around 9.00 am the PHM visited me. It was really good that she came. I had lot of things to ask her. She answered all of them with care. Then she examined me. She asked me whether I have any other discomfort. I trust her a lot!” Mi04	Mothers depend on us for all health activities. Even when the VOG asks to get a scan done, or admit to hospital or prescribe any drugs, the mothers ask us for advise and then proceed. NPC PHM
		Education sources	*Individual*	My sons’ class teacher said that he is doing well. I was so proud. He is only three and a half years. I was very happy.	
		Religious organizations		“The priest in the temple is very well known to my in laws. They are members of the temple committee. We went and asked for special blessings (“varu pirith”) that are offered for pregnant women. The priest asked me to come to the temple to have these blessings. Usually every pregnant woman in this area receive this during pregnancy.” NPC02	
		Micro-credit organizations		“I usually don’t go for these committees. Husband goes. Three women have to get together to get money. Earlier we took from a different company. This time the other two people wanted to get money again therefore we joined as help.” NPC01	“Usually the primi mothers do not participate in micro credit committees. But when mothers become older, having 3–4 children, take part in these. They are the people who want to collect equipment to house. Usually when children are older they have free time and they join these committees. It’s like an epidemic in the village. Husbands are laborers if they cannot have daily work they are unable to pay. Therefore conflicts occur in the family. It’s a burden to the society.”
		Police		“We went to town in the motorbike, my husband did not have license and we were not wearing helmets. The traffic police stopped us. But fortunately they let us go because I’m pregnant. I was so happy. Rarely that such good people exist in this community. It convinced me of the status given to a pregnant mother in this society.” R04	

^a^Frequently mentioned constructs

^b^Moderately mentioned constructs

^c^Constructs that were mentioned rarely in the diaries

**Table 2:** Framework analysis of structural constructs of social capital in pregnancy

Structural social capital construct	Definition	Descriptors	Level and type of Social capital	Selected verbatim/Description
Collective action^c^	The action taken by a group (either directly or on its behalf through an organization) in pursuit of members’ perceived shared interests. (Marshall 1998)	People get together in a tragedy	*Community Bonding*	“People get together when there is a problem to any one of the villagers. One day, our house was struck by lightening. We were not at home. When we arrived, all neighbors were already there.”
Informal social networks^a^	Web of relationships that people use to exchange resources and services (Cook 1982; Scott 1991; Wellman 1983). Informal networks are distinct from formal networks in that they are not officially recognized or mandated by organizations and in that the content of their exchanges can be work-related, personal, or social (Ibarra 1993).	Visiting or visited by neighbors/friends or relatives	*Individual* *Bonding*	Informal networks are dense, rich and are providing support in most of the communities.
Social Participation^a^	People’s social involvement and interaction with others that includes activities such as volunteering, making donations, participating in sports, and recreational activities are all forms of social participation.	Participation in religious activity	*Individual Bonding & bridging*	Religious participation has an individual as well as community variation. Generally in pregnancy the frequency of visits to religious institutions are high. Mothers participate in the weekly ritual of “Bodhipuja” & “Varu pirith” a religious blessings conducted by the Buddhist priests.
		Participation in cultural events	Individual/ community Bonding	“Today I was mostly in my mothers place. I helped my sisters to prepare sweets. It was a special occasion where we celebrate cooking the new rice from the paddy field. Although I felt tired I was happy to prepare sweets with my sister.” NPC06
		Participation in leisure activities	*Individual Bonding*	People do not use their leisure time properly. Instead they had few routine activities such as going for a bath in the tank, visiting the ancient city and taking children out into the field (“chena”) or park near by.
		Going to the fair This could be merged with above – its not different.	*Individual Bridging*	Sometimes pregnant women and most of the time their husbands visit the weekly fair held in the nearest town every Sunday.
		Visiting the city – same as above	*Individual* *Bridging*	Frequency of visiting the city varies in different communities. Some who are living near to the city visited daily. But the women in rural villages rarely saw the city. “There are mothers who visit the main city only 2–3 times per year” V PHM
Participation/membership in organizations^c^		Religious committees	Community/ individual Bonding/bridging	Generally, mothers are not active members of any of the committees except the women’s welfare societies. In some communities, these welfare societies are provide social support and help each other.
		Micro-credit committees		
		Womans’ welfare committees		
		Food committees		
		Funeral committees		
Links to government resources^b^ Access to health systems^b^		Meeting PHM	*Individual/community* *Linking & Bonding*	Every pregnant woman is under the care of a PHM appointed to that area. Pregnant women have a good relationship with the PHM and she provides emotional, instrumental and informational support regarding health.
		Meeting VOG	*Individual* *Linking*	Almost all mothers visit the specialist (VOG) at least once in their pregnancy. The routine scan recommended by the government antenatal care program receives high priority not only by mothers but also by their husbands. Many mothers visited the VOG at the private sector and others at the hospital clinic.
		Antenatal clinic/ sessions	*Community* *Linking*	All mothers routinely visit field antenatal clinics held in the community.
		Hospital	*Individual* *Linking*	Mothers visit the hospital once in a while for investigations & ultra sound scanning.
Access to educational institutions^c^		Visiting childs’ school	*Individual/community* *Linking*	Mothers who had children are concerned of the children’s education and communicate with class teacher and the school regularly.
Access to village leaders^c^		“Gramasevaka”	*Individual/ community* *Linking*	Mothers in this community rarely visit the community leaders.
		“Samurdhi” officer		
Access to other resources^a^		Telephone		Almost every mother have a mobile phone except one or two. It was a means of creating and sustaining social networks. Especially for mothers living away from their own relatives and in families where the husband works far away, the mobile phone act as a means of emotional support and care.
		Own vehicle		Not every family own a vehical. But most of them had access to three-wheelers in an emergency.
		Television/ radio		Many families have a television at home. Watching television reduced loneliness, offered informational support on health and some times created family cohesion.

^a^Frequently mentioned constructs

^b^Moderately mentioned constructs

^c^Constructs that were mentioned rarely in the diaries

During the next level of analysis, a thematic approach to identify and interpret the underlying mechanisms in which social capital constructs could affect health during pregnancy was used.

### Research rigor and quality control

#### *Streamlining data collection methods, tools* and analysis

All data collection methods and tools were designed according to accepted guidelines [35, 36]. Diaries, interview guides and note-taker forms were pretested. Quality of data collection was maintained by using checklists in all field visits. Interviewers were trained and supervised.

#### Triangulation

Triangulation minimizes bias due to chance associations and systematic biases due to a specific method in qualitative studies [37]. In this study three different data collection techniques - pregnancy diaries, diary interviews and in-depth interviews – and three different types of informants – pregnant women, PHMs and senior community dwellers were used to gather knowledge on social capital of pregnant women in each community. This gives a comprehensive insight into social capital in pregnancy, as expressed from different perspectives.

#### Respondent validation

In diary interviews, women were asked to reflect further on and agree or disagree with the particular types of social capital observed in their diaries. This gave researchers as well women a chance to elaborate on the written responses and obtain a more comprehensive data set. The situations of under reporting or over reporting were noted and clarified during diary interviews.

#### Reflexivity

Participants were asked to avoid behaviors other than natural in their day-to-day lives as change in behavior due to diary writing would result in invalid observations. Follow up interviews helped reveal if women had left out describing key daily social events from the diaries. Pre-determoned biased interpretations of the communities by the investigators were avoided using investigator diaries with memos throughout the study.

### Ethical considerations

Investigators followed these participants throughout the duration of diary writing. In the follow up interviews, women were counseled and arrangements were made to provide support. Contact details of the Principal Investigator (PI) were given to the participants and they were offered free counseling and or any medical advice throughout their pregnancy. Confidentiality and anonymity was maintained throughout the data collection, analysis and presentation.

Informed written consent was sought from all participants and participants were allowed to decline or withdraw from the study at any stage. Ethical clearance was obtained from the Ethics and Research Committee, Faculty of Medicine and Allied sciences, Rajarata University of Sri Lanka.

## Results

### Contribution from different qualitative methods

The diaries described the day-to-day relationships of pregnant women. The diary interviews were used to clarify doubts of the investigators and further explain already described relationships. The in-depth interviews with key informants were used to observe social capital of pregnant women in particular areas in their own point of view.

As framework approach was conducted, initially the results were explanatory, explaining how already identified dimensions and constructs manifest in pregnancy. The exploratory aspects of the study was propitious in identifying the real life descriptors of each construct, identifying new constructs and formulating a new framework for the association of social capital to pregnancy outcome.

### Dimensions of social capital in pregnancy

Diaries included detailed descriptions of both cognitive and structural social capital dimensions. The women described high levels of bonding and low levels of bridging social capital in pregnancy. Linking social capital was commonly observed in diaries as a means of accessing health but only rarely for other services (e.g. education, employment or political aspects). There were individual and inter-community variations in some dimensions, but high intra-community similarities were observed with regard to social participation, neighborhood trust and in domestic cohesion.

### Social capital constructs of pregnant women

During the framework analysis, ten cognitive constructs (Table 1) and five structural constructs of social capital (Table 2) were identified. For pregnant women in Sri Lanka, positive cognitive social capital was seen as more central than structural aspects.

### Cognitive constructs

Domestic and neighborhood cohesion were the most commonly expressed cognitive constructs among pregnant women in this study. As the social network of pregnant women was markedly limited to the micro community, domestic cohesion and, especially, relationship with the husband seemed to play a crucial role in determining social capital. Increased care and concern by the husband was a commonality in most of the communities during pregnancy. Most pregnant women, rather than female relatives and friends, saw the husband as the most trusted and closest person, and the person supposed to provide support during pregnancy. Intimacy in the wife-husband relationship seemed to have central role on social support received, as well as their sense of belonging.

Both positive and negative aspects (lack of) of social capital were observed, primarily in the constructs of domestic cohesion, neighborhood cohesion and social support. Many women experienced close and supportive cohesion within families in pregnancy despite living with in-laws.

Some Sri Lankan women, after marriage lived with their husbands’ parents. These women living away from their parents and closest relatives expressed loneliness in their diaries. In addition, these narratives describe that such loneliness is felt more deeply when there is conflicts with in-laws.

The study found, that social support of pregnant women was generally limited to support from the closest family members, a few trusted friends and from the PHMs. Trust in health institutions was high in almost all communities.

Investigators identified “*social contribution”* as a novel cognitive social capital construct. It was defined as “an individual’s obligation to act for the benefit of the society”: household responsibilities, contributing to religious or other cultural events and village organizations, and taking responsibility for the education of young. These responsibilities made pregnant women feel engaged and satisfied.

### Structural constructs

High density in structural social capital within the micro communities (families and close neighborhoods) was observed. Group membership was not common in pregnant women - such as membership of voluntary community groups except in health committees at the antenatal clinics. However, participation in religious activities was high in some communities. Almost all women in the study had a mobile phone, through which social relationships were maintained when husband or family was away.

### Social capital and its pathways to health in pregnancy

**Fig. 2:**
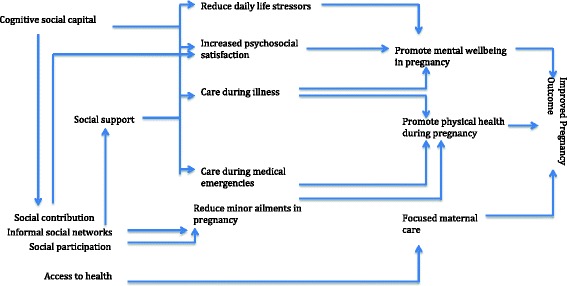
Social capital and hypothesized pathways to health in pregnancy

Four different pathways (themes) by which social capital could influence health in pregnancy were identified (Fig. 2).

#### Informal social networks and social participation as a means of reducing minor physical ailments in pregnancy

In many communities, pregnant women visited their neighbors, friends and relatives frequently. This helped them to get rid of minor physical symptoms that are commonly encountered in pregnancy (such as nausea, vomiting, backache, headache and abdominal discomfort) [49].

*“My relatives brought me food today. We all had dinner together. I was very happy. It made me forget all my bodily ailments” NPC02*

*“All pregnant women share their experience in bearing children. Every one see how their sisters, cousins and aunts go through pregnancy, have children and how they manage day-to-day activities. Therefore they do not complain about minor ailments.” PHM GA*

#### Cognitive social capital as a means of promoting mental wellbeing

In this study it was clear that cognitive social capital constructs including domestic cohesion, neighborhood cohesion, sense of belonging and reciprocity had a positive effect on the mental wellbeing of pregnant women. In particular, the husband’s role was mentioned frequently by all women.

### Domestic cohesion; husband’s role

The relationship with husbands played a major role in a woman’s life during pregnancy. Sri Lankan society is a patrilineal society. Participants indicated that when husbands demonstrated care for pregnant women, they felt protected and loved, buffering the sometimes-challenging social relationships with in-laws or neighbors. This was especially true when residence pattern was virilocal (Table 1).

*“My husband brought me fresh milk today as I had a food craving. He thinks about me a lot after I became pregnant.”* Med 03

*“I went to the hospital with my sister. My husband frequently phoned her and asked about my condition during the hospital visit. In the evening he came home and eagerly asked about the scan (ultrasound scan of the fetus). The doctor told me everything was fine, I said. He blessed me to spend the rest of my pregnancy safely. I’m happy. As I’m pregnant he cares a lot and sees to everything.”* Mi 02

Most women were very emotionally dependent on their husbands. When husband lived away from the house for employment or when relationships were temporarily disrupted due to alcohol or domestic conflicts, the pregnant women described poor mental wellbeing including a sense of social insecurity, loneliness and stress (Table 1).

### Domestic cohesion; family

Pregnant women in nuclear, as well as extended families, seemed to be emotionally influenced, positively as well as negatively, by the family cohesion. At times when family cohesion was supportive, women felt highly appreciated.

*"All people at home are awaiting for the great day! I'm very happy that my child is gifted with such a lovely family. He is very lucky" NPC01*

When family cohesion was disrupted due to conflict, pregnant women expressed great sadness and emotional distress. Conflicts with in-laws and living away from her parents were often mentioned as causing stress.

*“Today was a sad day for me. My husband got angry with me. We were supposed to go to my parent’s home at seven months of gestation (usually women go to their parents home for delivery). But I suggested leaving at sixth month. My husband became angry due to this and didn’t speak with me for two hours. After that he spoke. I love to be with my own family. I miss them a lot. The only person close to me in this family is my husband. So when he scolds me I’m so upset.”*

#### Neighborhood cohesion

Closely-knit neighborhoods were an asset for mental wellbeing of pregnant women. It provided the feeling of security and being cared for.

*"Every one in the neighborhood loves and cares about me. I'm very relaxed. This community is good, I would be able to deliver my child with good mental wellbeing." NPC01*

Being away from home and meeting friends (parents of other children) was also mentioned by women as reducing stress.

*"Today I took the child to pre-school English class. There I met other parents. We are friends. There are two friends who are pregnant as well. One of the parents had brought mango pickle. We tasted it until the class was empty. It’s joyful to spend time like this rather than staying alone at home. It makes me happy!"*

In situations where a mother felt anxious, low mood or lonely, a reciprocal and cohesive neighborhood was a protective factor for mental wellbeing.

*“Today was a very sad day for us. I woke up in the early morning and prepared food for my husband to take when he leaves (for work). My mother also helped me. My aunt (lokuamma) brought us fresh milk. As my husband is leaving, the grandfather (kiriaththa) came and tied a “pirith” string (a piece of string which is blessed with Buddhist chanting that is tied around a persons’ wrist which is meant to provide blessings). My uncle also came for the occasion. My husband left very sad. In the afternoon two nearby aunts came to see me with sweets. In the evening my mother, father and sister phoned me.” Med 03*

*“When I’m alone at home I feel very lonely. Therefore I’m used to visit my aunt or other neighbor all the time. It relieves loneliness and makes me happy.” V2*

Interestingly, pregnant women did not express feelings of insecurity, even if they felt lonely. Even though people are not emotionally close, the culture in the village was to always offer help when someone is in trouble. Women had the idea that some one will be there to help in an emergency.

##### Social support to enhance both physical and mental wellbeing

Most pregnant women received instrumental and emotional support through their micro-networks including close family and neighbors as described above. The well-established public health system, through primary health care workers, provided additional support on health.

### Instrumental support

Support to conduct household chores had an influence on the health of mothers, especially when they were ill. Six mothers reported episodes of illness during diary writing. Women in extended families always had support with household work when they were ill. Neighbors also helped by offering to cook meals. Mothers in nuclear families had difficulty in coping during illness, especially when there was not neighborhood cohesion.

*“By that time I was very ill. Because of the wound foot I had enlarged glands. My whole leg was swollen and it was difficult for me to walk. It was so painful. With all this, I helped my child to do the homework given by the school. Today there is a private class held at my place for children. Eleven children come for this. I was burning with high fever. I could not get up from bed. There was no one (to help). My husband goes out from the house to work. Only my child and myself live in our house. Luckily, the parents who brought their children to the class offered me great support. I would be helpless otherwise?” Mi01*

Availability of financial support ensured care during emergencies.

*"Although I had a problem with fetal movements, we did not have money to channel a doctor (means; go to a private practitioner). But when I told this to my mother she gave me money, which was a big relief" NPC06*

Absence of financial support was a factor that predisposed stress. (See Table 1-instrumental support -V04).

### Emotional support

Having a person close to share feelings was a rewarding method of stress reduction. However, despite strong micro-networks few mothers mentioned having a close person, with whom they could share their innermost feelings of happiness and grief. This ‘close person’ was an aunt, sister-in-law or mother-in-law.

*"A very close person visited us today. It was a great pleasure to see her. She is an aunt who is by my side - at times of joy and misery. She has brought dinner for me too" NPC02*

#### Linking social capital experienced through health services to promote maternal health during pregnancy

In the preventive care system, mothers received care, both through home and clinic visits. The clinic (nine or more visits during pregnancy) and domiciliary visits by the PHMs (one visit for each trimester or more) were conducted according to the scheduled guidelines of the Ministry of Health. Curative and specialized care was delivered at public private hospitals by desire or need.

### The public health midwife

All mothers expressed great trust in their PHMs and described them as the most accessible source of information on health during pregnancy.

*"The public health midwife visited around 10.30am. She examined me. She told me that my child is well and have turned to birth position now. She also inspected the bag I have prepared to take to the hospital. She told me the procedures that I will go through when I'm admitted to hospital" NPE02*

*“Our PHM is very good. She’s so close to us. She advise us on everything” R03*

Expectant mothers perceived extra health security, if the PHM was living close by.

*“Today I was not well with backache. I had numbness in one leg. The PHM told me not to worry. She said it’s because I’m a little overweight. She’s living next door. Therefore we are not afraid of illnesses. Can ask anything.” P03*

### Antenatal clinics and sessions

All pregnant women in this study regularly attended antenatal clinics. Clinics were situated nearby in all neighborhoods.

“*Having the antenatal clinic is a big relief. I can learn a lot. Everyday is a new experience. Today I cooked early morning and set off to the antenatal clinic with my husband. My blood was taken for investigation and they also did a dental examination.” Med03*

Pregnant women expressed trust in all types of the health personnel working in the clinic, namely the PHM, the public health nursing sister (PHNS) and the MOH. Clinics also provided informational support.

*“The MOH talked about the impact of the living environment on health during pregnancy. She told us lot of things. We learnt a lot” Mi04*

The antenatal clinics gave the opportunity of meeting other pregnant women and thus strengthening additional social capital. First time mothers also benefited from listening to the experiences of mothers who had children before.

The antenatal sessions, that were recently introduced in Sri Lanka, created an opportunity for pregnant women and their husbands to get relevant health related information. The PHMs mentioned that women benefited from antenatal care as it provided an opportunity to improve health through participation of their husbands. Antenatal clinics, classes and related committees seem were the only means of effective group membership during pregnancy.

### Meeting the specialist; visiting obstetrician & gynecologist (VOG)

Pregnant women were referred to the VOG for the routine dating scan or if there were any risk factors discovered by the primary health providers. They met experts at both government and at private health care institutions. Whether there was a clinical need or not, meeting a specialist and getting reassurance on the progress of the pregnancy was a high priority for most of women in the study, except for those of low socio-economic status.

*“I met the obstetrician only twice in private health care institutions. Local health personnel told me that it is not compulsory to see him. My husband took me to see the VOG. He said that my child is well, and gave a date to be admitted at the hospital.” NPC01*

## Discussion

Although the role of social capital and health is studied extensively, this is the first paper to discuss social capital in pregnancy in-depth. Social capital constructs commonly assessed in large-scale population surveys [50, 51] such as community trust, group membership, voting or political engagement and collective action did not seem to be important indicators during pregnancy in this sample of rural Sri Lankan women. Instead, social networks of pregnant women community were restricted to family members, close friends and relatives. These close networks were rich and dense, with cognitive (domestic cohesion, neighborhood cohesion, sense of belonging and perceived social support) and structural bonding (informal social networks).

This qualitative study identified a variety of descriptors for social capital constructs in pregnant women (Tables 1 and 2). Most of the descriptors were unique to pregnancy. The typical descriptors used do not appear sensitive enough to capture the “real life” means of social capital in pregnancy. These new descriptors could be used to formulate or adapt tools that measure social capital in pregnancy. Sensitivity of these descriptors should be further tested through quantitative studies.

There is debate currently between individual and community social capital. This study denotes that even in homogenous communities, individual variations in social capital (domestic cohesion, social support and social participation) exist that can affect physical and, especially, mental wellbeing during pregnancy. Among the limited studies done on social capital and pregnancy, a multi-level cohort study conducted in Brazil showed that low individual social capital rather than low neighborhood social capital to be associated with poor self-rated health during pregnancy [13]. The narratives in this study show how poor neighborhood social capital is buffered by family cohesion and how poor family can be buffered by the marital relationship.

This study focused mainly on individual social capital. It was observed that pregnant women are not the group to ask/observe on community social capital as generally in rural areas and in the Sri Lankan culture pregnancy is considered as a socially immobilized time period. Some aspects of collective action were observed within health committees and in cultural events. However collective efficacy and informal social control supported pregnant women (Tables 1 and 2). Pregnant women did not express insecure feelings, even if they felt lonely. They had the belief that even if there was no close person, people will always be there to help in an emergency.

Social capital could influence both physical and mental health of pregnant women through different mechanisms. Minor physical ailments, although ignored often by the health personnel, can result in significant loss of productivity [11]. This study showed frequent informal social networks and social participation that mediate through neighborhood bonding, can relieve minor physical symptoms.

Studies suggest that cognitive rather than structural social capital is more associated with health [9]. In this study, we found this to be true, with cognitive social capital playing a major role for health in pregnancy. This study showed how social capital was enhanced during pregnancy, starting from husbands providing additional care, neighbors and relatives offering foods and people at social gatherings paying special attention, respecting and talking to the pregnant women about her future. All work through trust, harmony and support to enhance mental wellbeing of a pregnant woman.

This study showed, that diaries of pregnant women with poor domestic and neighborhood cohesion and kinship demonstrated loneliness, stress and poor mental wellbeing. Hence, it is important to identify pregnant women living in neighborhoods with poor social capital. Early identification of reduced cognitive social capital among pregnant women could prove to be an effective way of promoting mental wellbeing.

Social support has been found to improve pregnancy outcomes [15, 52]. Whether social support acts through improvements in psycho-social resources or by improving socio-economic conditions is debated [15]. This study clearly observed that the social support received by pregnant women improved psycho-social resources. Improving socio-economic conditions as an independent factor for promoting wellbeing should not as a result of these findings.

Sri Lanka has a strong health system and maternal care is a high priority. This study shows that the health system is the only organization through which linking social capital comes into play in pregnancy. Pregnant women have trust in the grass root level health care workers (PHM) who provides all types of social support (informational, instrumental and emotional) during pregnancy. Women routinely attend antenatal clinics and also benefit from care offered by specialist obstetricians. The study, therefore, underlines that the Sri Lankan health system acts as a means of strong linking social capital to improve the health of pregnant women. It was observe that the “social support perspective” and “political economy approach” [25] could affect health in pregnancy. It was also observe that the Sri Lankan public health system, by providing universal health coverage, has been able to overcome the major health related disadvantages that are created through socio-economic inequalities of pregnant women. Discussing community differences of social capital is beyond the scope of this paper as the aim was to obtain a holistic picture of social capital among pregnant women in Sri Lanka.

Although the negative influences of poor or absent social capital on maternal health were observed, situations where women had negative experiences through received social capital was not seen in this study. Ill health behaviors, such as smoking, that could be initiated through presence of social capital, were scarce among pregnant women especially in these rural communities. Participation in groups other than for religious or health aspects were low among these pregnant women.

Although strict procedures were maintained for trustworthiness and quality assurance, and sought diversity in selecting study participants, there may have been bias in the selection process, as the study district has a large population with contextual diversity. Although illiterate participants were not found, the literacy level and intellectual ability of the participants were not equal which might have influenced the study. Most of the social capital constructs (especially cognitive constructs) are interrelated.; therefore, classifying the data into separate constructs was sometimes difficult and overlapping constructs were sometimes observed. Eight study communities were included in this study; although, findings are not generalizable they likely reflect conditions in most rural communities in Sri Lanka including minority ethnic communities. Nevertheless, the health related social capital presented here may be unique to the strong public health networks with free health services. Capturing the different types of social capital would have been a problem if investigators used a structured diary. Therefore the diaries were unstructured purposefully but allowed the participants to write each and every social relationship they came across during each day. Hence it was assumed that the finding of networks limiting to micro communities (family and neighborhood) is the real life situation among rural pregnant women.

## Conclusions

In conclusion, investigators propose two analytical inductive approaches for further research. The first approach is to describe the social capital constructs in pregnancy (based on Fig. 1). It was found that domestic and neighborhood cohesion are the most commonly expressed social capital constructs in pregnancy among rural Sri Lankan women. Current social capital tools do not reflect this, and may therefore not fully capture social capital of pregnant women. Future researchers can use these constructs and their descriptors when studying social capital in similar settings.

**Fig. 3:**
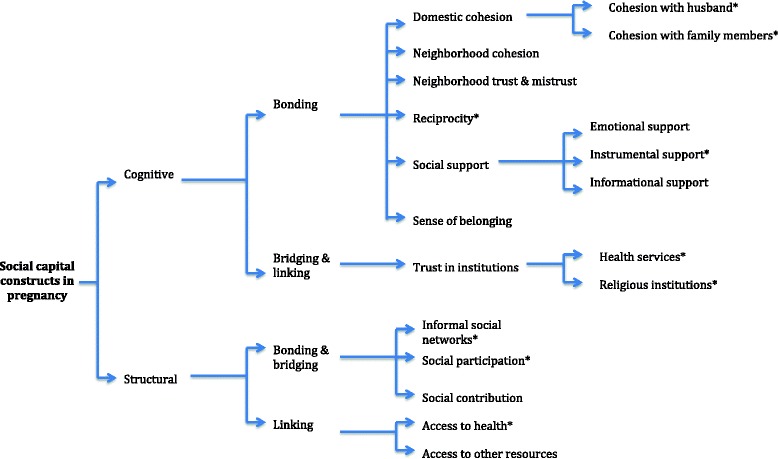
Social capital dimensions in pregnancy

The second approach (illustrated in Fig. 3) illustrates the hypothesized links between social capital and wellbeing during pregnancy, which acts through social support perspective and political economy approach in the initial deductive framework. The above identified social assets act through psychosocial support mechanisms to improve health during pregnancy. A woman could be under risk if these micro community networks are disturbed. Researchers, policy makers and program planners could use these findings to investigate social capital as a major determinant for positive outcome of antenatal care. The political economy context, by providing means of linking social capital and facilitating bonding mechanisms among women even at peripheries through both public and curative health systems have been able to buffer the effects of socio-economic inequalities that could affect health during pregnancy. We recommend further quantitative research using appropriate and sensitive tools to measure social capital in pregnancy, as this could be a cornerstone in understanding how to further reduce maternal morbidity and mortality in LMICs.

## Abbreviations

GA: Gambirigaswewa and Aluthgama
HICs: High Income Countries
LMICs: Low and Middle Income Countries
Me: Medawachchiya
Mi: Mihinthale
MOH: Medical Officer of Health
NPC: Nuwaragam Palatha Central
NPE: Nuwaragam Palatha East
P: Padaviya
PHM: Public Health Midwife
PHNS: Public Health Nursing Sister
R: Rajanganaya
V: Vilachchiya
WHO: World Health Organization
